# *Alternaria* Mycotoxins in the Hemp (*Cannabis sativa* L.) Food Chain

**DOI:** 10.3390/toxins18050218

**Published:** 2026-05-05

**Authors:** Terenzio Bertuzzi, Lorena Schiavi, Federico Siboni, Roberta Battaglia, Paola Giorni, Domenica Iraci Capuccinello, Massimo Montanari

**Affiliations:** 1 Department of Animal, Food and Nutrition Science (DIANA), Faculty of Agriculture, Food and Environmental Science, Università Cattolica del Sacro Cuore, 29122 Piacenza, Italy; lorena.schiavi01@icatt.it (L.S.); federicosiboni40@gmail.com (F.S.); roberta.battaglia1@unicatt.it (R.B.); 2 Department of Sustainable Crop Production (DIPROVES), Faculty of Agriculture, Food and Environmental Science, Università Cattolica del Sacro Cuore, 29122 Piacenza, Italy; 3 Research Center Plant Protection and Certification (CREA DC), Council for Agricultural Research and Analysis of the Agricultural Economy (CREA), 40128 Bologna, Italy; domenica.iracicapuccinello@crea.gov.it; 4 Research Center Cereal and Industrial Crops (CREA-CI), Council for Agricultural Research and Analysis of the Agricultural Economy (CREA), 40128 Bologna, Italy; massimo.montanari@crea.gov.it

**Keywords:** *Alternaria* toxins, hemp seeds, hemp oil, defatted cake

## Abstract

Hemp seeds and derived products were recently re-evaluated in the food sector, thanks to their high nutritional value and absence of gluten. This increasing diffusion required to investigate the occurrence of mycotoxins, in particular of *Alternaria* toxins (ALTs), has been uncovered at high levels in previous work. An integrated approach was involved in this study. First, *Alternaria* spp. incidence and ALTs were determined in hemp seeds harvested in different fields during 2024 and 2025 and the influence of meteorological conditions and of varieties was evaluated. Then, their distribution in hemp oil and defatted flour was studied after a cold pressing process of naturally contaminated hemp seeds, finding a high percentage, over 85%, in hemp cake. Finally, a small survey was conducted on different hemp products intended for direct human consumption, confirming the risk of contamination in seeds, flour and derived bakery products.

## 1. Introduction

In recent years, hemp (*Cannabis sativa* L.) received renewed interest in the agri-food sector, establishing itself as a multifunctional crop capable of combining environmental sustainability, technological innovation and nutritional enhancement. After a period of marginalization due to cannabinoid restrictions, hemp has been re-evaluated as a strategic resource for the ecological transition and the circular economy. Its adaptability, low input requirements and the valorization of different fractions make it interesting for modern agriculture. In recent years (from 2015 to 2022), the production of hemp increased in the EU from 94,120 to 152,820 tonnes (a 62.4% increase) [1]. In Italy, the cultivation of hemp varieties included in the EU variety register and with a maximum D9-THC (tetrahydrocannabinol) content of 0.3% is permitted [2]. For hemp seeds intended for human consumption, a maximum level for D9-THC equivalents of 3 mg/kg was set by the European Commission Regulation [3]. In the food sector, hemp seeds and derived products, such as oil, flours, protein products and bakery products, are receiving significant growth in the market, thanks to their high nutritional value and absence of gluten. The seeds are a balanced source of essential polyunsaturated fatty acids, with an optimal ratio of ω-6/ω-3 as well as containing proteins of high biological quality rich in essential amino acids [4]. This growing diffusion requires investigation for all the aspects related to food safety, considering also the occurrence of mycotoxins. Different fungal diseases, belonging to *Aspergillus*, *Fusarium*, *Penicillium* and *Alternaria* genera, can infect *Cannabis sativa* plants [5,6,7,8,9,10,11,12,13] and the presence of their different mycotoxins can occur in seeds. Mycotoxins are implicated in several human health problems owing to their stability during food processing and have also been found to cause a serious health risk to animals consuming contaminated feed. A limited number of studies have investigated the contamination by mycotoxins in hemp seeds and derived products [14,15,16,17]. These contaminants can develop at different stages of the hemp chain, from cultivation to storage and to processing. Recently, a worrying occurrence of high levels of *Alternaria* toxins (ALTs) was found in hemp seeds harvested in an experimental field during the years 2018–2022 [18]. These compounds represent an emerging group of mycotoxins, detected in various plant-based foods but not yet regulated in Europe. Alternariol (AOH) and alternariol monomethyl ether (AME) can induce cell cytotoxicity and cause cell apoptosis; moreover, they can trigger mutagenicity in either bacterial cells or mammalian cells and induce primary DNA damage in vitro and in vivo. TeA inhibits newly formed proteins from the ribosomes, reducing cell viability across mammalian cell lines; TeA cytotoxicity effects involved liver damage, gastrointestinal hemorrhage, cardiovascular collapse and mortality in various animal species [19]. In 2022, the European Commission published the Recommendation 2022/553 [20] which indicates benchmark levels for AOH, AME and TeA in certain foods, including sesame and sunflower seeds and sunflower oil; no levels for hemp-based products were reported. Considering the risk of high ALT contamination levels in hemp-based products and the lack of information related to the conditions which can influence their production, the aim of the present work was to investigate the occurrence and distribution of *Alternaria* mycotoxins in the hemp food chain, with particular attention to seeds and the main derived products, such as flour and oil. The study involved an integrated approach: First, determination of mycotoxins in hemp seeds collected across two years (2024 and 2025) from several fields located in different Italian regions was carried out, evaluating the influence of variety and of meteorological conditions between flowering and harvest. Then, their distribution in oil and defatted flour obtained from cold pressing processes of hemp seeds was determined. Finally, their occurrence in different commercial derived products, including whole and hulled seeds, flours, bakery products, oils, protein products and herbal tea, was surveyed. This approach allowed us to assess the level of contamination at the different stages of the hemp chain.

## 2. Results

### 2.1. Alternaria Toxins Occurrence in Hemp Seeds Harvested in Field

All hemp seed samples were contaminated with TeA; the contamination levels of ALTs were very different among the varieties and the years (Table 1). Considering the varieties (four) collected in both years, a significant difference was found among the years (2024 more contaminated than 2025) and the varieties (Table 2). The contamination observed in the hemp seed samples collected in 2024 and 2025 can be explained by the different meteorological conditions that characterized the two years, in particular with reference to the period between flowering and harvesting, which generally occurs in August and September, except for the early-maturing Carifit1p line. Overall, the year 2024 was characterized by intense rainfalls in August and especially in September, while in 2025 rainfalls were less incisive and more distributed. In Rovigo, the year 2024 showed a moderate rainfall in August (33.6 mm), but high in September (104.6 mm); moreover, heavy rainfall for the period occurred in June (174.4 mm). In 2025, the same field showed a high value in August (93.6 mm), but very low in September (26.4 mm). In Anzola Emilia (BO), 2024 was particularly rainy in both August (58.4 mm) and September (174.0 mm) and also in May (123.2 mm), while, in 2025, the values were much lower with limited rainfall (25.8 and 22.0 mm in August and September, respectively). In Osimo (AN), precipitations in 2024 were generally average and limited in August (11.2 mm), but an exceptional event occurred in September, with 322.4 mm of rain. In 2025, on the contrary, rainfall was more distributed: 99.8 mm in August and 40.4 mm in September. Finally, the high ALTs contamination values found in hemp samples grown in the Monterotondo field in 2025 were probably due to the late harvesting date (late October) and to the widespread precipitations registered in summer–autumn (62.8, 91.8 and 88.8 mm from August to October); in previous months, the precipitations in both years were average for the period. The trend of relative humidity confirmed more favorable conditions for fungal development in 2024. On the other hand, average temperatures are comparable between the two years, with maximum values between 24 and 28.5 °C in July–September. These data showed how intense precipitations in the final months of the crop cycle (August–September) can slow down the natural drying of the hemp seeds and favor the development of *Alternaria* spp. and the subsequent production of mycotoxins. Regearding the genotype aspect, the comparison among the five varieties/lines grown in both years (Futura 75, Codimono, Carifit1p, Carifit2t and P1) showed significantly higher contamination levels of TeA in Carifit 2t, while Carifit1p showed less contamination (*p* ≤ 0.05) (Table 2). This difference was probably due to the different harvest time, since Carifit1p is an early variety and was harvested in late July or early August.

Different locations were considered for Codimono, which was the only hemp variety cultivated in different locations in both years. However, no statistical significant difference was found among locations.

### 2.2. Fungal Incidence

Incidence of total fungal presence did not show statistically significant differences among the different hemp varieties even if, considering a specific genus, Codimono and P1 showed higher contamination by *Alternaria* spp. and Carifit1p was the most resistant to these fungi (Table 2). However, some varieties showed to be more susceptible to Alternaria species infection, showing a significantly higher presence of these kind of fungi (Codimono and P1) (Table 2).

### 2.3. Distribution of Alternaria Toxins During Cold Pressure Process

**Figure 1:**
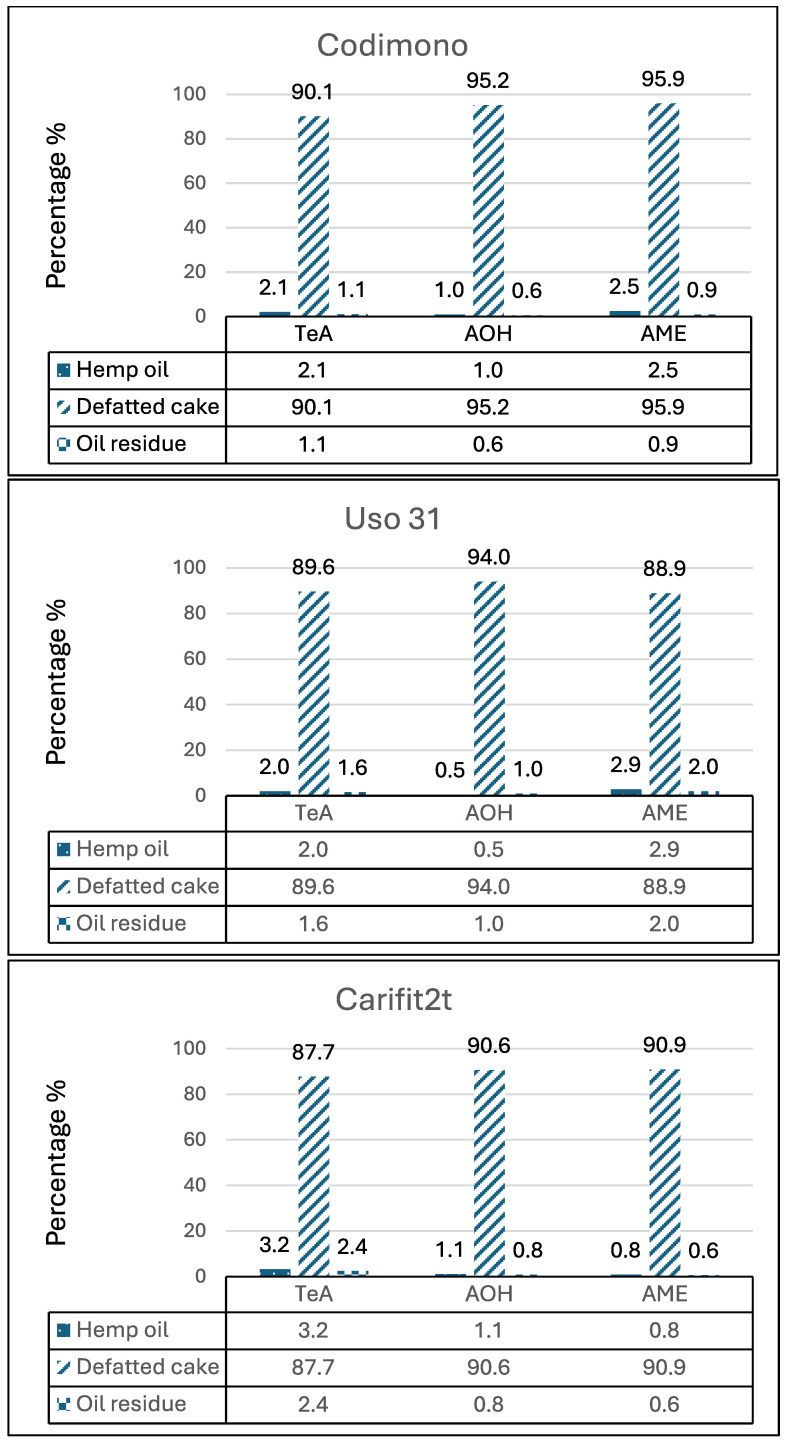
Percentage distribution of TeA, AOH and AME in oil, defatted cake and oil residue after cold pressing process.

To investigate the distribution of ALTs in oil and defatted flour (used for different bakery products), four batches of seeds of different varieties (Codimono, Uso 31, Carifit1p and Carifit2t) were subjected to cold pressure processes. The levels of TeA, AOH and AME were determined in seeds and in the main fractions obtained from the process: oil, defatted cake and residual solid lipidic fraction. The results clearly showed that the highest concentrations of ALTs were always found in defatted cake, while the lipid components (oil and oil residue) showed very low values, confirming the low affinity of ALTs for lipid matrices (Figure 1); because of the low contamination level in seeds of the Carifit1p variety, no ALTs were detected in oil and oil solid residue.

The average percentage distribution in defatted cake was 89.1 ± 1.3%, 93.3 ± 2.4% and 91.9 ± 3.6% for TeA, AOH and AME, respectively. The average mass balance of the three processes, calculated as the ratio ((amount toxin in seeds)/(amount toxin in oil + flour + residue))*100 was 93.3 ± 0.1%, 94.9 ± 2.2% and 95.1 ± 3.7% for TeA, AOH and AME, respectively. Finally, two samples of defatted cake (Carifit2t and Carifit1p) were sifted (450 µm) eliminating almost all of the integral part and analyzed for ALTs determination; no reduction of the contamination was observed.

### 2.4. Survey of Alternaria Toxins in Commercial Hemp-Based Products

The study also included the analysis of a selection of hemp-based products collected in several Italian markets, with the aim of assessing the actual level of contamination of the products destined to direct human consumption. This small survey covered different hemp-based food matrices, including commercial seeds, flour, bakery products (crackers, biscuits, breadsticks and bars), protein products and herbal teas. Commercial products showed significantly lower levels of contamination than raw seeds harvested in the field. TeA was the most detected mycotoxin, AOH was detected only in 1 sample and AME were always below the LOD (Table 3); it is possible to note that the hulling process significantly reduced ALTs contamination in seeds. No contamination was found in protein products and in hemp tea. This trend indicated a substantial reduction of these toxins along the processing chain. Considering the benchmark value of 1000 µg/kg for TeA in sunflower seeds reported in the Recommendation 2022/553, only one sample would have exceeded this value.

## 3. Discussion

The high contamination levels of ALTs found in hemp seed samples harvested in several experimental fields confirmed the findings of Lanzanova et al. [14], who found very high incidence of these mycotoxins in hemp seed samples collected in the same experimental field during the 2018–2022 period. It is known that *Alternaria* spp. can develop easily in humid conditions and that the production of ALTs can be stimulated by rapid changes in temperatures due to rainfall [21]. In the abovementioned study [18], the abundant rainfall occurred in 2018 during the phases of flowering, and pre-harvest induced a delay in the harvest time favoring the presence of *Alternaria* spp. and the production of ALTs; moreover, humid conditions occurred during the post-harvest in which hemp seeds are generally ripened in air to decrease the moisture level, also contributing to increased contamination. Finally, the study of Lanzanova et al. [18] reported that the Carmaleonte variety was the most contaminated by ALTs. In this study, the abundant rainfall occurred in 2024 at harvest time, generally in September, in all the experimental fields, which highlighted how this meteorological condition can favor ALTs production. Considering that heavy rainfall becomes common in this period of climate change, an early line (Carifit1p) was cultivated, harvested in the summer period, when rainfall is absent or very scarce in Italy. A significantly lower contamination rate was obtained, showing that the development of early varieties can limit the risk of ALTs contamination. As regards the other varieties, Carmaleonte was confirmed to be a sensible variety to ALT contamination, showing very high levels of contamination in 2024 even if harvested in early August; then, it was not cultivated in 2025. To our knowledge, no study reported in the literature evaluated the distribution of ALTs in hemp oil pressing processes. Our results are in line with those for other oilseeds (sesame and sunflower), showing that these mycotoxins tend to concentrate in the residual oilcake, rather than in the finished oil [22,23]. A recent survey of Zhou et al. [24] in several vegetable oils reported a widespread occurrence of different mycotoxins but at very low levels, including AME; the low contamination levels could be due to a low distribution of the main mycotoxins in vegetable oils. These findings have important operational repercussions, in particular for the production of flour and bakery products, which required accurate selection and cleaning steps, and constant monitoring as well as the use of hulled seeds when possible. The small survey confirmed that the risk of contamination in oil is very limited, such as in protein products and in herbal tea.

## 4. Conclusions

Hemp can be considered a useful crop for ecological transition; moreover, several positive bioproducts occur in seeds, indicated for both human and animal consumption. However, ALTs can frequently occur in seeds, and this study highlights the need to evaluate their occurrence in hemp seeds harvested in the field and to develop adequate solutions to reduce the strong influence of meteorological conditions at the harvest time, including possible post-harvest management. Further studies are necessary to verify resistance of hemp varieties to *Alternaria* spp. and ALTs in case of highly rainy weather conditions during the growing season and to formulate useful suggestions for farmers, keeping in consideration that early cultivations seem to have a lower incidence of infection by these fungi and mycotoxins. In line with EFSA recommendations for emerging food matrices, constant monitoring is needed in seeds and bakery products destined for direct human consumption.

## 5. Materials and Methods

### 5.1. In-Field Sampling of Hemp Seeds Samples

A total of 30 samples of hemp seeds destined for human consumption, belonging to 7 monoecious and 2 dioecious varieties, were harvested in 6 experimental fields located in 4 Italian regions (Emilia-Romagna. Veneto, Marche and Lazio) in 2024 and 2025, respectively. Meteorological data (total monthly rainfall (mm), average relative humidity (%) and average temperature (°C)) were collected using meteorological stations close to fields used for the trials. The collected samples were air dried and non-hulled; they were kept at −20 °C before milling (1 mm sieve) and analyzed on the same day for *Alternaria* toxins determination.

### 5.2. Cold Pressing Process

Four naturally contaminated samples of hemp seeds were subjected to a cold pressing process to obtain hemp oil and defatted cake. Starting from 15.5 kg of seeds, 14 kg of defatted cake, 1 L of oil and 0.5 kg of oil residue (solid lipidic phase) were obtained. All the samples were kept at −20 °C until the analysis.

### 5.3. Sampling of Derived Hemp-Based Products

Various commercial derived hemp-based samples (*n* = 25) were collected in different retails located in Northern Italy in 2025; in particular, the samples were: hulled and non-hulled seeds (3 + 3), flour (3), bakery products (5), hemp oil (5), hemp protein products (3) and herbal tea (3). Solid samples were milled (1 mm sieve) and kept at −20 °C until the analysis. Hemp oil samples were kept at +4 °C.

### 5.4. Fungal Incidence Determination

Fifty kernels of hemp seeds were disinfected in 1% sodium hypochlorite solution (NaClO) for 2 min followed by 80% ethyl alcohol solution for another 2 min and then rinsed with sterile distilled water for 5 min. When dried, hemp seeds were transferred to Petri dishes containing Potato Dextrose Agar (PDA) and incubated at 25 °C (12 h light/12 h dark) for 7 days. At the end of incubation, the incidence of infected seeds was calculated by evaluating the developed fungal colonies counted out of 50 (the total number of hemp kernels). The same procedure was followed to determine the incidence of the *Alternaria* spp. genera; the identification at genus level was based on observations with binocular microscope (×40). The analysis was conducted in triplicate for each sample.

### 5.5. Analysis for Alternaria Toxins Determination

*Alternaria* toxins (TeA, AOH and AME) were extracted with a mixture acetonitrile: water 80 + 20 *v*/*v* for 60 min using a rotary shaker; after filtration on folded filter paper, an aliquot (0.2 mL) of the extract was transferred in a vial, diluted with 0.6 mL of deionized water and injected in an HPLC-MS/MS system (Thermo-Fisher, San Jose, CA, USA); for TeA determination, the extract was again diluted in a range between 10 and 50 times. ALTs were separated on a HSS-T3 RP-18 column (5 µm particle size, 150 × 2.1 mm, Waters) with a mobile-phase acetonitrile–water (both acidified with 0.2% formic acid) in gradient mode as follows: from 30:70 to 70:30 in 5 min, then isocratic for 4 min; gradient to 30:70 in 1 min and reconditioning for 6 min. The ionization was carried out using an ESI interface (Thermo-Fisher) in positive mode as follows: spray capillary voltage 4.5 kV, sheath and auxiliary gas 35 and 12 psi, respectively, vaporizer temperature 200 °C, temperature of ion transfer tube 270 °C. The fragmentation ions were: 125, 139 and 153 *m*/*z* for TeA (parent ion 198 *m*/*z*); 128, 185 and 213 *m*/*z* for AOH (parent ion 259 *m*/*z*); 128, 184 and 258 *m*/*z* for AME (parent ion 273 *m*/*z*); collision gas (Argon) 1.5 psi, collision energy among 16 and 38 V. The LOD and the LOQ were 3 and 10 µg/kg for TeA, 1 µg/kg and 2 µg/kg for AOH and AME.

### 5.6. Statistical Analysis of Data

Incidence of fungal presence was arcsine-transformed and mycotoxins content was log-transformed before statistical analysis [25]. All data obtained were subjected to univariate analysis of variance (ANOVA) using the generalized linear model (GLM) procedure, and significant differences between means were confirmed using Tukey’s test. The data were tested for normality (Shapiro–Wilk test) prior to the analysis. The statistical package IBM SPSS statistics 29 (IBM Corp., Armonk, NY, USA) was used for data analysis.

## Figures and Tables

**Table 1:** Alternaria toxins concentration (µg/kg) in hemp seeds harvested in experimental fields in 2024 and 2025.

*2024*					
Variety/line	Location	Harvest data	TeA	AOH	AME
Uso 31	Rovigo (RO)	23 September 2024	290,676.2	5713.0	8067.2
Codimono	Rovigo (RO) *	23 September 2024	127,580.9	403.2	629.6
Codimono	Rovigo (RO)	23 September 2024	156,061.1	116.0	78.3
Futura 75	Rovigo (RO)	23 September 2024	197,884.2	271.6	317.1
Carifit1p	Rovigo (RO)	13 August 2024	28,037.0	<1	<1
Carmaleonte	Rovigo (RO)	4 September 2024	23,728.7	48.1	<1
P1	Anzola Emilia (BO)	15 October 2024	88,228.8	900.8	2010.3
Carifit1p	Anzola Emilia (BO)	30 July 2024	5608.7	<1	<1
Carifit1p	Anzola Emilia (BO)	27 November 2024	8687.0	<1	<1
Codimono	Bentivoglio (BO)	12 September 2024	91,117.9	408.5	868.2
Carmaleonte	Osimo (AN)	28 August 2024	34,329.4	64.0	138.0
Codimono	Osimo (AN)	21 October 2024	13,141.6	<1	<1
Carifit2t	Osimo (AN)	26 September 2024	29,466.1	3.8	3.7
Codimono	Monterotondo (RM)	21 September 2024	55,900.7	<1	<1
Carmaleonte	Monterotondo (RM)	5 August 2024	81,308.6	<1	<1
Mean			84,983.9	890.8	1533.0
*2025*					
Variety/Line	Location	Harvest data	TeA	AOH	AME
Fibranova	Rovigo (RO)	17 September 2025	2585.3	<1	<1
Codimono	Rovigo (RO)	17 September 2025	10,760.8	4.3	<1
Futura 75	Rovigo (RO)	17 September 2025	8068.9	<1	4.3
Carifit1p	Anzola Emilia (BO)	8 August 2025	1637.4	2.3	7.2
Carifit1p	Anzola Emilia (BO)	8 August 2025	1892.7	<1	<1
Carifit1p	Anzola Emilia (BO)	8 August 2025	2064.5	<1	<1
P1	Anzola Emilia (BO)	30 September 2025	838.4	<1	<1
Carifit1p	Anzola Emilia (BO)	13 November 2025	8272.3	26.8	21.1
Carifit1p	Osimo (AN)	31 July 2025	18,987.2	4.1	8.4
Carifit2t	Osimo (AN)	29 September 2025	18,242.2	2.4	<1
Codimono	Osimo (AN)	16 September 2025	5436.3	1.3	3.9
Carifit2t	Osimo (AN)	26 September 2024	14,256.1	3.2	3.5
Carifit1p	Osimo (AN)	31 July 2025	1581.3	<1	<1
Estica	Jesi (AN)	15 September 2025	2755.8	1.3	8.6
Codimono	Monterotondo (RM)	20 October 2025	47,128.0	105.7	99.8
Mean			10,647.8	16.9	19.6

* The underlined varieties were tested in the same location in both years.

**Table 2:** Analysis of variance (ANOVA) of total fungal presence (%), *Alternaria* spp. incidence (%) and *Alternaria* toxins contamination in the different varieties and in the two years considered in the study (2024 and 2025). Different letters mean significant differences according to Tukey’s test (** *p* ≤ 0.01; * *p* ≤ 0.05; ns: not significant).

Factor	Alternaria Incidence (%)		Total Fungi Incidence (%)		TeA (µg/kg)		AOH (µg/kg)		AME (µg/kg)	
(A) Variety	*		ns		*		ns		ns	
Carifit1p	4.0	B	87.2		8530	B	4		4	
Carifit2t	7.2	AB	90.5		238,542	A	31		19	
Codimono	12.7	A	75.7		64,399	AB	126		204	
Futura 75	7.0	AB	91.3		102,977	AB	136		161	
P1	9.6	A	82.2		44,534	B	450		1005	
(B) Year	ns		*		**		*		*	
2024	11.8		79.2		94,948	A	186	A	341	A
2025	7.55		94.1		24,091	B	14	B	12	B
AxB	*		ns		ns		ns		ns	

**Table 3:** Concentration levels (µg/kg) of TeA, AOH and AME in commercial hemp-based products.

	TeA	AOH	AME
Whole seeds	545.3	<1	<1
Whole seeds	1250.0	<1	<1
Whole seeds	640.3	<1	<1
Hulled seeds	41.1	<1	<1
Hulled seeds	63.6	<1	<1
Hulled seeds	<10	<1	<1
Flour	2282.1	<1	<1
	320.5	<1	<1
	614.0	<1	<1
Bakery products	73.8	<1	<1
	84.3	<1	<1
	155.1	<1	<1
	1524.1	14.5	<1
	618.0	<1	<1
Oil	15.8	<1	<1
	8.9	<1	<1
	8.9	<1	<1
	<10	<1	<1
	<10	<1	<1
Protein products	<10	<1	<1
	<10	<1	<1
	<10	<1	<1
Tea	<10	<1	<1
	<10	<1	<1
	<10	<1	<1

## Data Availability

The original contributions presented in this study are included in the article. Further inquiries can be directed to the corresponding authors.
